# Assessing effectiveness of a community based health insurance in rural Burkina Faso

**DOI:** 10.1186/1472-6963-12-363

**Published:** 2012-10-19

**Authors:** Sennen Hounton, Peter Byass, Bocar Kouyate

**Affiliations:** 1 Department of Epidemiology, Centre MURAZ, 2054, Avenue Mamadou KONATE, 01 BP 390, Bobo-Dioulasso, Burkina Faso; 2 Department of Public Health and Clinical Medicine, Umea Centre for Global Health Research, Umea University, Umea, Sweden; 3 Ministry of Health, Ouagadougou, Burkina Faso

**Keywords:** Effectiveness, Community based health insurance, Universal health coverage

## Abstract

**Background:**

Financial barriers are a recognized major bottleneck of access and use of health services. The aim of this study was to assess effectiveness of a community based health insurance (CBHI) scheme on utilization of health services as well as on mortality and morbidity.

**Methods:**

Data were collected from April to December 2007 from the Nouna’s Demographic Surveillance System on overall mortality, utilization of health services, household characteristics, distance to health facilities, membership in the Nouna CBHI. We analyzed differentials in overall mortality and selected maternal health process measures between members and non-members of the insurance scheme.

**Results:**

After adjusting for covariates there was no significant difference in overall mortality between households who could not have been members (because their area was yet to be covered by the stepped-wedged scheme), non-members but whose households could have been members (areas covered but not enrolled), and members of the insurance scheme. The risk of overall mortality increased significantly with distance to health facility (35% more outside Nouna town) and with education level (37% lower when at least primary school education achieved in households).

**Conclusion:**

There was no statistically significant difference in overall mortality between members and non-members. The enrolment rates remain low, with selection bias. It is important that community based health insurances, exemptions fees policy and national health insurances be evaluated on prevention of deaths and severe morbidities instead of on drop-out rates, selection bias, adverse selection and catastrophic payments for health care only. Effective social protection will require national health insurance.

## Background

**Figure 1:**
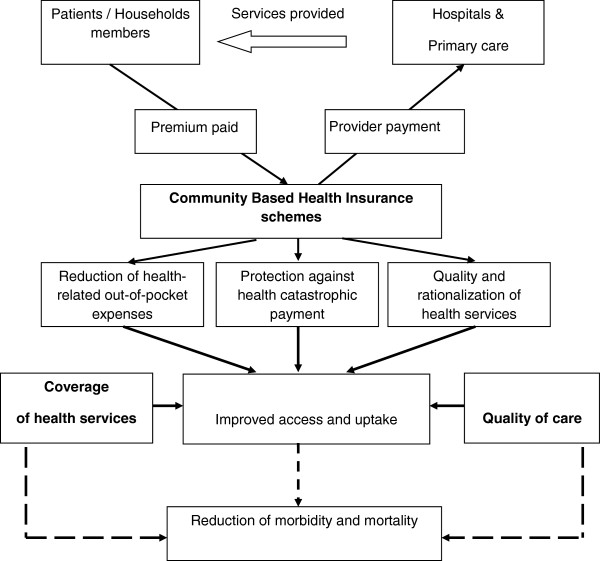
**Community Based Health Insurance framework (adapted from Bennet S **[16]**).**

Community based health insurance schemes are based on the premises of risk-pooling and community solidarity to risks of falling sick and are conceptually designed to provide financial protection and reduce out-of-pocket for care for health [1,2]. By providing this financial protection, community based health insurance schemes could potentially increase access and utilization of health services (Figure 1), and thus increase antenatal care and institutional delivery [3]. Indeed, in the endeavour of protecting the community against a brutal and unaffordable cost of illness, the community based health insurances schemes need to make sure that there is no adverse selection or moral hazard [4] in terms of enrolment and use of health services, or drop-out [5]. The evidence on effectiveness of community based health insurances schemes has been mixed. Penetration rates (enrolment rates) of CBHI schemes are often low, ranging between 3% to 5% of the targeted population and rarely 10% [6-8]. Carrin G et al. [9] reported that the achievements of CBHI in terms of revenue collection, pooling of resources and purchasing of services were very modest, and the effectiveness of CBHI in terms of quality of care or representativeness of members with regards to the targeted population was weak or inexistent [10-12]. Also, there has been report of an enrolment bias towards the least poor subset of the population, and that the level of enrolment and use of the services by the most poor was not great enough to compensate for pre-existing inequities in access [3]. Some of the premises of the CBHI schemes are to protect against catastrophic payments and to ensure access to quality care and therefore it is important for policy makers to identify most effective ways to ensure effectiveness and sustainability of community based health insurance schemes [13]. The review of previous experiences of CBHI schemes points to a selection bias in most cases, with poorest people less likely to enroll as compared to least poor [7,14,15], and to challenges regarding achieving equity in access and uptake of services. The discussions about effectiveness of community based health insurance schemes are thus often around enrolment rates, drop-outs, selection bias, cost-recovery after a serious illness, or catastrophic health payments and barely about the overarching goal of utilization of quality health services and reduction of mortality and morbidity. This paper sought to contribute to the debate of effectiveness of community based health insurance schemes by assessing the effects of the Nouna community based health insurance scheme in improving utilization of quality health services and reduction of mortality and morbidity.

## Methods

The surveys and data extraction were conducted from April to December May 2007.

### Study site and populations

**Figure 2:**
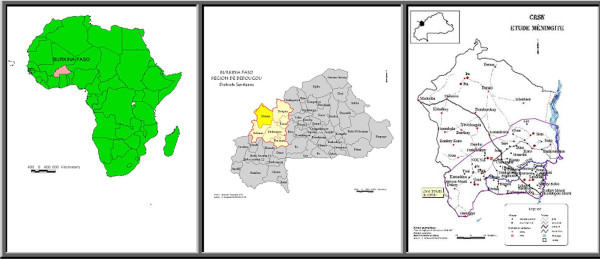
Location of Nouna DSS, Burkina Faso (Photo credit: Dr Bocar Kouyate).

The study site is Nouna health district (Figure 2), a remote and rural health district situated in the North West of Burkina Faso. The area is characterized by dry weather with a mean annual rainfall of about 800 mm resulting in dry savannah vegetation. In early 1990s, a Demographic Surveillance System (DSS) was established by the Nouna Health Research Centre. The original DSS area covered 39 villages (~population about 26 000 inhabitants) and has been progressively extended to cover 58 villages and Nouna town, with a population of about 72 000 people. The density of population is about 35 individuals per square km. The population is distributed in roughly 9,500 households and composed of 65% of a rural people and 35% of Nouna semi-urban town people. The population is essentially young with children less than 15 years of age representing about 48% of the total population, and only 6.2% above 60 years of age. The inhabitants are mostly subsistence farmers and/or cattle keepers. Illiteracy is extremely high, over 80%.

### The Nouna community based health insurance (CBHI) scheme

**Figure 3:**
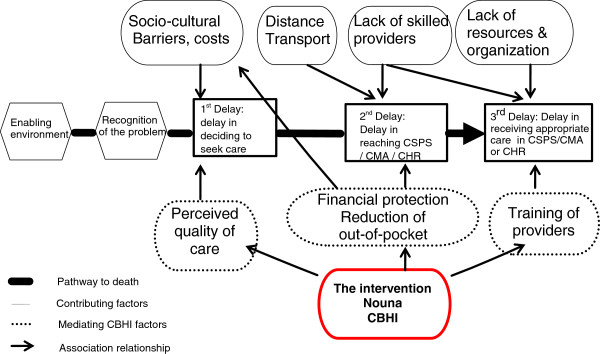
Conceptual model of the Nouna community based health insurance scheme.

The Nouna CBHI was launched in 2004 and was developed by the Nouna Health Research Centre in collaboration with the University of Heidelberg (Germany) as an operational research to study how to improve community access and uptake of health services and how to meet the need of the poor within Nouna health district. The scheme was progressively introduced since 2004 using a stepped-wedged design after almost two years of preparation. In 2004 only one third of the targeted areas was covered by the intervention, followed by another third in 2005, and the last third in 2006. The preparation phase involved structural meetings with key stakeholders, administrative clearance, several studies on barriers and facilitators of implementing such strategies, scenarios of benefit packages, valuation of health outcomes willingness-to-pay for the scheme, population revenue and affordable premium and has been extensively described elsewhere [6,17-20]. The benefit package included the minimum package of primary care services available in the district including antenatal care, laboratory exams, hospitalization fees, and transportation for emergencies. Members of the scheme are individual and families from covered health areas within the demographic surveillance system (DSS) who paid the enrolment fee (0.4 USD), and annual premium (3 USD per individual 15 years and older and 2 USD per individual less than 15 years of age). By improving perceived quality of care, by reducing out-of-pocket and by ensuring financial protection reduces the overall delay (mainly first and second), the Nouna CBHI is expected to improving utilization of quality health services and reduction of mortality and morbidity (Figure 3). Thus, we can assume if the Nouna CBHI is effective one could expect a higher utilization of health services (including but not limited to antenatal care, institutional delivery, malaria, etc.) among household members of the Nouna CBHI versus non-members. The improved utilization of health services could result in a lower mortality risk (screening, early diagnostic, access to emergency treatment) in members compared to non-members which we sought to investigate after adjusting for important determinants such as education, distance to health services and household asset ownership.

### Data collection and analysis

Data were extracted from longitudinal household surveys at the Nouna Demographic Surveillance Site. The household survey sample size was 1,504 households at the time of the study, half of which were from Nouna town and the other half from surrounding villages; data were extracted on household characteristics, births, deaths and age at death, distance from village to health centers and to Nouna district hospital. In addition, a prospective survey was administered to women with experience of delivery during the last 12 months prior to the survey (April – May 2007) and data were collected on place of delivery, membership in the Nouna Community Based Health Insurance, age of the mother, anemia [21], average distance from village to health centre, and assets ownership. Anemia was selected as this morbidity has not been investigated at the time the study was designed. Descriptive statistics and regression analyses (logistic regression and Poisson regression) were performed to assess the association of overall mortality, utilization of health services, and institutional delivery by membership to the Nouna CBHI adjusting for important covariates. We used a Poisson regression with computed person-time of all deaths (person-time of years spent before death in the DSS, since the DSS started) to investigate whether there is any mortality advantage (lower mortality risk) to any sub group and by any of the selected covariates, and assess whether there is an overall lower mortality risk among members versus non-members and by selected covariates. This method was used because the distribution of deaths is probably skewed and the person-time variable consists of non-negative integers. The dependent variable was the person-time of all deaths in the Nouna DSS and membership in the Nouna CBHI, educational level, age, asset ownership, place the explanatory variables. Dummy variables were created reflect the time spent in the scheme.

### Ethical consideration

Study was approved by ethical review boards of Centre MURAZ and the Nouna Health Research Centre (Burkina Faso).

## Results

### Descriptive analysis of the study populations

Table 1 describes the characteristics of the households included in the Nouna panel household survey by enrolment status in the Nouna CBHI scheme and by the selected covariates (education, place, perceived quality of care, asset ownership). The two groups are comparable with respect to mean age of head of household (49.6 for households not members versus 50.8 for households members, t-test p = 0.148). There were significant differentials in enrolment in the Nouna CBHI scheme by utilization of health services and by covariates. There was a 14 percentage point difference (85.4 - 71.4) in the utilization of health services between members and non-members. Similarly there were 20.6 (59.3 – 38.7), 23.1 (63.2 – 40.1), and 18.7 (34.3 – 15.6) percentage point differences between members and non-members for people with at least primary level of education, people living in Nouna town, and assets ownership respectively.

**Table 1:** Descriptive characteristics of populations by enrolment status (from household survey, 1504 household)

**Characteristics**	**Members**	**Not members**	**P-value**
	**N (%)**	**N (%)**	
**Use of health services**			0.000
- Did not use	53 (14.6)	281 (28.6)	
- Have used	310 (85.4)	700 (71.4)	
**Education**			0.000
- None	148 (40.7)	602 (61.3)	
- At least primary school level	216 (59.3)	380 (38.7)	
**Place**			0.000
- Nouna town	230 (63.2)	394 (40.1)	
- Nouna villages	134 (36.8)	588 (59.9)	
**Asset ownership**			0.000
- Most poor	3 (0.8)	235 (23.9)	
- Second quartile	35 (9.6)	235 (23.9)	
- Third quintile	85 (23.4)	198 (20.2)	
- Fourth quintile	116 (31.9)	161 (16.4)	
- Least poor	125 (34.3)	153 (15.6)	

P-values are the level of significance on the Pearson chi-square test between enrolment and covariates. There is no difference by age or sex between members and not members.

### Effects on utilization of health services

Using a descriptive and a logistic regression analysis to assess the effectiveness on utilization of health services adjusting for explanatory variables (Tables 2 and 3). There remains a statistically significant association between membership of the Nouna CBHI scheme and the utilization of health services after adjusting for the covariates. Members are 2.23 times more likely to use health services compared to non-members. Similarly, people living in Nouna villages (versus Nouna town), people with at least primary level education are 2.09, and 1.60 times more likely to use health services compared to their counterparts respectively. For asset ownership, the results are difficult to interpret. People in the second and third quintiles made greater use of health services whilst for the fourth and fifth (least poor) quintiles statistical significance was not reached.

**Table 2:** Utilization of health services by enrolment status in Nouna CBHI scheme and by household characteristics (Household survey, 1504 household)

**Household characteristics**	**Did not use services**	**Used services**	**P-value**
	**N (%)**	**N (%)**	
**Education : None**			0.000
- Members	25 (11.4)	123 (23.3)	
- Non members	195 (88.6)	406 (76.7)	
**Education : At least primary level**			
- Members	28 (24.4)	187 (38.9)	
- Non members	86 (75.4)	294 (61.1)	
**Place: Nouna town**			0.002
- Members	37 (20.9)	192 (43)	
- Non members	140 (79.1)	254 (57)	
**Place : Nouna villages**			
- Members	16 (10.2)	118 (20.9)	
- Non members	140 (89.8)	446 (79.1)	
**Perceived quality of care: Low**			0.000
- Members	7 (8.5)	91 (27.5)	
- Non members	75 (91.5)	240 (72.5)	
**Perceived quality of care: High**			
- Members	44 (17.6)	219 (32.3)	
- Non members	206 (82.4)	458 (67.7)	
**Asset ownership : Most poor**			0.162
- Members	20 (36.4)	104 (46.8)	
- Non members	35 (63.6)	118 (53.2)	
**Asset ownership : Least poor**			0.280
- Members	0 (0)	3 (1.7)	
- Non members	66 (100)	169 (98.3)	

**Table 3:** Logistic regression of utilization of health services by enrolment status and by covariates

**Dummy**	**B**	**Std. Err**	**Wald**	**Sig**	**Exp(B)**	**95% Conf. Interval**	
**variables**						**Lower**	**Higher**
Constant (α)	2.256	0.239	89.07	0.000	9.528	-	-
CBHI (1)	- 0.802	0.179	19.9	0.000	0.448	0.315	0.637
Nouna town (1)	0.739	0.152	23.58	0.000	2.094	1.554	2.822
PQOC* (1)	- 0.393	0.151	6.79	0.000	0.675	0.502	0.907
Education (1)	- 0.476	0.140	11.51	0.01	0.622	0.472	0.818
Asset ownership							
Qunitile (2)	- 0.592	0.247	5.74	0.017	0.553	0.341	0.898
Quintile (3)	- 0.713	0.219	10.61	0.01	0.490	0.319	0.753
Quintile (4)	- 0.257	0.216	1.425	0.233	0.773	0.507	1.18
Quintile (5)	−0.081	0.223	0.133	0.715	0.922	0.596	1.42

PQOC: Perceived Quality of Care.

Chi-square (H-L): 7.275 P-value 0.507.

-2LL: 1413 R-squared : 0.063.

CBHI_0 = reference group, not members.

CBHI (1) = comparative group, members.

Education 0 = reference group, no education.

Education (1) = comparative group, at least primary school level education.

PQOC 0 = reference group, low perceived quality of care.

PQOC (1) = comparative group, high perceived quality of care.

### Effect of Nouna CBHI on mortality

The question we had was whether or not members experience a lower mortality risk compared to non-members. After adjusting for distance to district hospital, household size, and education level of head of household (Table 4), there was no statistically significant difference in overall mortality between groups of individuals whose households could not have been members in the scheme (because their area was yet to be covered by the scheme), individuals not members but whose households could have been members (areas covered by Nouna scheme), and individuals members in the Nouna CBHI scheme. There was a lower mortality risk to members by year of enrolment although there was no ‘dose-response’ with years spent in the scheme. However, there were significant differences by covariates in mortality risk. The risk of overall mortality among households from 0km up to 35 km away from Nouna town was 35% higher than the risk of overall mortality among the reference group (Nouna town) and the risk of overall mortality among households more than 35 km from Nouna town was 50% higher than the risk of overall mortality in Nouna town.

**Table 4:** Poisson regression of mortality status (person-time of all deaths) by enrolment status and by covariates (distance, place of residence, household size, educational level), Nouna DSS, Burkina Faso

	**Robust**				**95% Conf. Interval**	
**Died**	**IRR**	**Std. Err**	**Z**	**P>|z|**	**Lower**	**Higher**
Enrolment_1	0.97	0.11	- 0.22	0.826	0.78	1.22
Enrolment_2	0.87	0.14	- 0.86	0.390	0.65	1.18
Firstyear	0.74	0.07	- 3.19	0.001	0.62	0.89
Secondyear	0.70	0.08	- 3.28	0.001	0.57	0.87
Thirdyear	0.72	0.09	- 2.54	0.011	0.55	0.93
Distance_1	1.54	0.09	6.78	0.000	1.36	1.75
Distance_2	2.01	0.14	10.39	0.000	1.76	2.30
Size_1	0.75	0.04	- 6.11	0.000	0.68	0.82
Education_1	0.73	0.04	- 5.15	0.000	0.65	0.82
Education_2	0.53	0.09	- 3.59	0.000	0.38	0.75
Person-time	(exposure)					

(Std. Err. adjusted for 16397 clusters in id individuals).

Number of obs = 52382 Wald chi2(10) = 247.23.

Log pseudolikelihood = − 9375.1604 Prob > chi2 = 0.0000.

Enrolment_0 is the reference group and represents the group of individuals whose households could not have been members in the scheme because their area was yet to be covered by the scheme.

Enrolment_1 = represents the group of individuals whose households could have been members (areas covered by Nouna scheme), but were not.

Enrolment_2 = represents the group of individuals whose households were members, and their areas were covered by Nouna CBHI scheme, and.

Person-time is defined as time spent up to death within the Nouna DSS from inception throughout 2007.

Enrolment_0 is the reference group (deaths before the insurance scheme) and additional dummy variables were created (firstyear, secondyear, and thirdyear) for time spent in the scheme to assess any “dose-response” relationship.

Firstyear = year 1 of the Nouna CBHI (2004–2005).

Secondyear = year 2 of the Nouna CBHI (2005–2006).

Thirdyear = year 3 of the Nouna CBHI (2006–2007).

For distance, dummy variable were created in relation to Nouna town where is the district hospital (and not distance to health centers because life saving skills and equipment are mainly at the district hospital).

Distance_0 = reference group for distance to district hospital (Nouna town).

Distance_1 = group of households not in Nouna town and less than 35 km from the district hospital.

Distance_2 = group of households at more than 35 km from district hospital.

(Size) Household size_0 = reference group (household size 1–10).

(Size) Household size_1 = group of households with more than 10 members.

Education_0 = reference group for educational level (none).

Education_1 = primary school level.

Education_2 = secondary level or more.

Similarly, the risk of overall mortality among households whose head of household had at least primary school level education was 37 % lower than the risk of overall mortality among the reference group (no education level) and the risk of overall mortality among households whose head of household had at least secondary school level education was 88% lower than the risk of overall mortality among the reference group. Also, the risk of overall mortality within households covered by the scheme and members in the scheme was 88% lower than the risk of overall mortality among the reference group of households not members (because their areas were yet to be covered).

### Effects on anemia among women with recent deliveries

Table 5 presents levels of anemia by antenatal care visits and institutional delivery. There was a significant association between severe anaemia and antenatal care visits prior to delivery, and also an association between severe anaemia and whether or not a woman delivered at a health facility. Apart from the significant association, it is also interesting to note that there were significantly more severe anaemia cases in the group of women with no antenatal care visits compared to women with at least one antenatal care visit (13 versus 5 respectively), and significantly more severe anaemia cases in the group of women who had a home delivery compared to women who had an institutional delivery (220 versus 18 cases).

**Table 5:** Description of levels of haemoglobin by antenatal care visits, and institutional delivery Nouna household cost survey

**Haemogobin levels**	**At least one antenatal care visit**	**No antenatal care visit**	**Pearson’s chi-squared**
	**N (%)**	**N (%)**	
(Hg ≤ 7g/dl)	13 (5.6)	5 (27.8)	p value = 0.000
Hg › 7g/dl	220 (94.4)	13 (72.2)	
Total	233 (100)	18 (100)	
**Haemogobin levels**	Institutional delivery	Home Delivery	
	N (%)	N (%)	
(Hg ≤ 7g/dl)	3 (2.6)	15 (11.2)	P value = 0.008
Hg › 7g/dl	114 (97.4)	119 (88.2)	
Total	117 (100)	134 (100)	

## Discussions

It was not clear to us whether people used services more because they were enrolled or they were enrolled because they tended to use services more. Our results point to a selection bias in enrolment in the Nouna CBHI. This was confirmed by the mortality analysis. Although one may argue that CBHI alone may not reduce mortality rates, we can reasonably assume that, everything being equal, reduction of financial barriers could significantly reduce the delays in accessing emergency care and therefore reduce the likelihood of mortality. Our study found no “dose–response” between the number of years of exposure to the scheme (enrolment in the scheme) and the risk of overall mortality, and no significant association between risk of overall mortality and areas covered by the scheme. Groups of individuals living close to Nouna town and in less crowded households, and individuals whose heads of household were better educated experienced a lower mortality risk compared to their counterparts living further away from the district hospital (more remote villages), staying in more crowded households, and whose heads of household had no education respectively.

The observed differences in overall mortality may be due to selection bias (ie people members in the scheme tend to be better off compared to non-members in terms of educational background, asset ownership, birth cohorts, household size, distance to health services, etc.), and a possible selection bias in the step-wedge design. In fact, one may hypothesize that people living in areas covered initially may be better off compared to people living in areas covered by subsequent waves of implementation. It is not unusual when launching an intervention to begin where there is a better likelihood of success before rolling the intervention out; but then one should be cautious about any observed outcome as the results may be confounded by who the recipients were and the context of the intervention instead of the intervention itself [22].

Regarding the data about anemia, one would expect that a greater utilization of health services may translate into a greater likelihood of early detection of anaemia among pregnant women during antenatal care visits and newly delivered women. The early detections will thus prompt care which will subsequently result in the lower prevalence of anaemia cases. These results point to fewer cases of severe anaemia among women who delivered in health facilities and this may have various interpretations. It may be that the anaemia condition triggered the visits to health facilities, hence the high prevalence (absolute numbers) of severe anaemia in the antenatal care visit category. It may well also be that compared to women who delivered at health facilities, an anaemia condition among those who delivered at home is less likely to be diagnosed and to be treated, and hence the observed lower levels of haemoglobin in the post-partum periods in which the survey took place. Another possible explanation is that low quality of care given by traditional birth attendants during delivery that could result in less control of haemorrhage during or after delivery and hence lower levels of haemoglobin in the post-partum periods for women who did not deliver in facilities. Regardless of the actual causal mechanisms, these findings confirm our assumption that a greater utilization of health services might translate into a greater likelihood of early detection of anaemia among pregnant and newly delivered women, and therefore better care and lower levels of anaemia. We could not find any difference in levels of haemoglobin by membership in the community based health insurance scheme. Finally, we could not find an association between delivery within an institution, antenatal care and membership of the community based health insurance scheme. This finding is puzzling because these associations are reasonable expectations using our conceptual framework and given that the Community Based Health Insurance scheme aimed to increase utilization of health services. However, the sample size (251 eligible women with delivery in the last 12 months before the survey), may well be too small to detect any effect, or maybe there are other factors that confound the relationship between enrolment in the Community Based Health Insurance scheme and utilization of health services.

### Limitations

The inferences made were based on data collected over 4 years ago, and the evolution of health outcomes may have changed. Also financial barrier is only one bottleneck in access and uptake of quality care, and the limited recall period since the launch of the Nouna CBHI (2–3 years) may not be sufficient to observe a change in outcomes. Nonetheless, change in health outcomes should the standard practice in evaluating any health intervention.

## Conclusion

There was an independent and significant association between utilization of health services and membership of the scheme even after adjusting for education level, place of residence, and asset ownership, and even with the evidence of selection bias at both the design stage and in the membership. There was no observed association between an institutional delivery, antenatal care, levels of haemoglobin of women and membership of the community based health insurance scheme. Regarding mortality, even without actually being part of the Nouna CBHI scheme, households that could have been members (being located in areas covered by the scheme) experienced a 50% lower mortality rate compared to households not members because their areas were yet to be covered by the scheme, which point to a selection bias. We observed no significant association between memberships of the Nouna community based health insurance scheme and overall mortality within households. Given the usual low enrolment rates and the selection bias in most community based health insurance scheme, it is important for future assessments of community based health insurance schemes, exemptions fees schemes or national health insurance to focus on actual prevention of mortality and morbidities [23,24] and not only on adverse selection, drop-out rates and catastrophic payments for health care or utilization of services.

## Competing interests

The authors declare that they have no competing interests.

## Authors' contributions

SH designed and conducted the study, and drafted the manuscript. PB contributed to data analysis and interpretation. BK contributed to the design and the review of the manuscript. All authors read and approved the final manuscript.

## Authors' information

Sennen Hounton is a medical epidemiologist with expertise in maternal and newborn health, health systems and economic evaluation. Sennen Hounton is an MD (Benin), MPH in Epidemiology (University of Oklahoma, USA) and a PhD in Public Health from University of Aberdeen, (Scotland, UK). He was a Senior Research Fellow with Immpact (Initiative for Maternal Mortality Program Assessment). He is currently Maternal Health Technical Adviser at the United Nations Population Fund (New–York), and serves as Scientific and Technical Advisor on the WHO Alliance for Health Policy and System Research Scientific and Technical Advisory Committee.

Peter Byass is Professor of Global Health, and Director of the Umeå Centre for Global Health Research (UCGHR) in Sweden. UCGHR is a leading research centre, relating human health to epidemiological transition, life-course interventions, primary care, gender and climate. Professor Peter Byass works with his team all around the world on research that tries to improve peoples’ health and lives.

Dr Bocar Kouyate is public health and health system research expert and Special Advisor to the Ministry of Health, Burkina Faso. He was the founding Director of the Nouna Health Research Center and the Director of the National Malaria Research Center in Burkina Faso. Dr Bocar Kouyate is a member of numerous scientific and advisory technical committees in health system research.

## Pre-publication history

The pre-publication history for this paper can be accessed here:

http://www.biomedcentral.com/1472-6963/12/363/prepub
